# Urological cancer related to familial syndromes

**DOI:** 10.1590/S1677-5538.IBJU.2016.0125

**Published:** 2017

**Authors:** Walter Henriques da Costa, George Jabboure, Isabela Werneck da Cunha

**Affiliations:** 1 Departamento de Urologia, AC Camargo Cancer Center, São Paulo, SP, Brasil;; 2 Department of Pathology, Department of Urology and Department of Oncology, Johns Hopkins University - Baltimore, Maryland, United States;; 3 Departamento de Patologia, AC Camargo Cancer Center, São Paulo, SP, Brasil

**Keywords:** Urinary Tract, Syndrome, Neoplastic Syndromes, Hereditary

## Abstract

Cancer related to hereditary syndromes corresponds to approximately 5-10% of all tumors. Among those from the genitourinary system, many tumors had been identified to be related to genetic syndromes in the last years with the advent of new molecular genetic tests. New entities were described or better characterized, especially in kidney cancer such as hereditary leiomyomatosis renal cell carcinoma (HLRCC), succinate dehydrogenase kidney cancer (SDH-RCC), and more recently BAP1 germline mutation related RCC. Among tumors from the bladder or renal pelvis, some studies had reinforced the role of germline mutations in mismatch repair (MMR) genes, especially in young patients. In prostate adenocarcinoma, besides mutations in BRCA1 and BRCA2 genes that are known to increase the incidence of high-risk cancer in young patients, new studies have shown mutation in other gene such as HOXB13 and also polymorphisms in MYC, MSMB, KLK2 and KLK3 that can be related to hereditary prostate cancer. Finally, tumors from testis that showed an increased in 8 - 10-fold in siblings and 4 - 6-fold in sons of germ cell tumors (TGCT) patients, have been related to alteration in X chromosome. Also genome wide association studies GWAS pointed new genes that can also be related to increase of this susceptibility.

## INTRODUCTION

Hereditary cancer syndromes account for 5 to 10% of all cancers and are characterized by a high predisposition to develop tumors. Most are autosomal dominant diseases characterized by a familial clustering of early onset cancer. Over the last two decades, clinical molecular genetic testing for diagnosing the underlying molecular alteration responsible for one of the known cancer predisposition syndromes has increasingly become available. This has enabled the implementation of effective screening guidelines as well as the detection of asymptomatic carriers among family members. Currently, there are approximately 100 genes known to predispose to one or more forms of cancer in carriers of germline pathogenic mutations.

**Figure 1:**
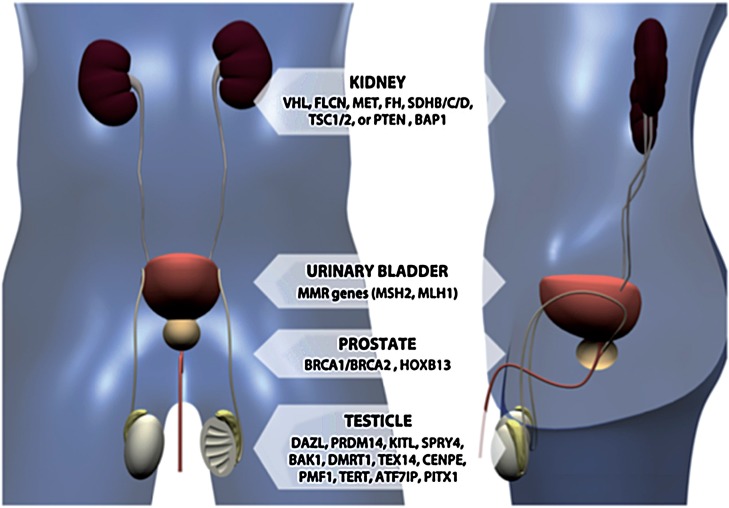
Main genes related to urological malignances linked to genetic syndromes.

Tumors of urinary tract can be a manifestation of several different genetic syndromes (Figure-1). In this review we focused on organ based tumors and highlight the principal subtypes and clinical morphological parameters that should be recognized in other to further investigations.

## MATERIALS AND METHODS

We comprehensively searched the MEDLINE and Cochrane databases on 4 July, 2015. Search terms included the USA National Library of Medicine’s Medical Subject Headings (MeSH): familial prostate cancer, hereditary prostate cancer, familial kidney cancer, hereditary kidney cancer, kidney cancer syndromes, familial testis cancer, hereditary testis cancer, Lynch Syndrome, hereditary urothelial carcinoma, familial urothelial carcinoma, P53 mutation, TP53 mutation, Li-Fraumeni Syndrome. Both free text and MeSH search for key words were used. Data from a period of 22 years, from 1993 to 2015, were included in the search.

## RESULTS

### Kidney

Renal cancer accounts for 2 - 3% of all malignant disease in adults. The incidence of renal cancer is rising at a rate of approximately 2.5% per year in the USA. Hereditary renal cell carcinoma (RCC) represents 5% to 8% of kidney neoplasms. Family history, clinical manifestations common in familial syndromes, bilateral or multifocal tumors and relatively young patients age (up to 46 years old) are findings that should suggest hereditary RCC.

Several hereditary RCC syndromes have been described, including von Hippel-Lindau (VHL), Birt-Hogg-Dube´ (BHD), hereditary papillary renal cell carcinoma (HPRC), hereditary leiomyomatosis RCC (HLRCC), succinate dehydrogenase kidney cancer (SDH-RCC), tuberous sclerosis (TS), and Cowden syndrome (CS). These syndromes have been shown to be associated with germline mutations in VHL, FLCN, MET, FH, SDHB/C/D, TSC1/2, or PTEN genes, respectively. Recently, germline mutations of BAP1 gene have been described as a possible finding that leads to hereditary predisposition to RCC.

VHL disease is the first described and the most common hereditary renal cancer syndrome. It represents an autosomal dominant inherited disorder caused by germline mutations in the VHL gene (1). Affected individuals are at risk for the development of tumors in several organs, including multifocal clear cell RCC, pancreatic cysts, neuroendocrine tumors, pheochromocytoma, retinal angiomas and central nervous system (CNS) hemangioblastomas. It affects approximately 1 in 36.000 live births worldwide. The VHL gene is a tumor suppressor gene located on the short-arm of chromosome 3 (3p25). Loss of heterozygosity (LOH) of VHL is commonly found in clear cell RCC (ccRCC) in patients with VHL syndrome as well as those in the sporadic setting (2). The syndrome specific lesions differ in mean age of onset, frequency and in the underlying type of germline mutation (3). In fact, the disease is classified into types 1 and 2 based on the presence of pheochromocytoma. The second group is further subdivided into 2A, 2B and 2C depending on the presence of RCC (Table-1).

**Table 1:** Type and characteristics of VHL genetic syndrome.

Type 1	VHL loss or mutation that affects the protein folding	Haemangioblastoma Renal Cell Carcinoma Low risk of phaeocromocytoma
Type 2A	VHL missence mutation	Haemangioblastoma phaeocromocytoma Low risk of Renal Cell Carcinoma
Type 2B	VHL missence mutation	Haemangioblastoma Renal Cell Carcinoma Phaeocromocytoma
Type 2C	VHL missence mutation	Phaeocromocytoma only

Approximately two thirds of patients present multiple renal cysts and RCC patients with VHL disease can develop up to several hundred cysts and tumors. RCC occurs with an age dependent frequency ranging from 25% up to 70%. The lifetime risk for developing RCC is 25% to 45%, and when renal cysts are included, the risk rises to 60% (4). Much controversy exists regarding the biological characteristic of VHL - associated tumors. Some groups have reported that the growth rate of VHL associated tumors is slower than that of sporadic tumors whereas others could not confirm such findings (5, 6). Life expectancy of patients is around 50 years old and RCC is the leading cause of death (3).

HLRCC is a hereditary syndrome characterized by the presence of cutaneous and uterine leiomyoma. Renal tumors have been identified in approximately one-third of HLRCC families. Though less penetrants, renal tumors usually present aggressive behavior (7). Affected individuals harbor a germline heterozygous loss-of-function mutation of the Krebs cycle enzyme, fumarate hydratase (FH) gene, which acts as tumor suppressor. Patients with HLRCC inherit a germline mutation of the FH gene as well as a wild-type copy. LOH at 1q43 is observed in up to 80% of patients, which suggests a biallelic inactivation of the FH gene, in a “two-hit” manner; one allele inactivated by the germline mutation and the other by LOH. The exact mechanism of tumorigenesis in HLRCC is unknown, but evidences suggest a pseudo - hypoxic pathway, similarly to the molecular mechanism in VHL - deficient kidney cancer.

HPRC is an autosomal dominant hereditary syndrome in which patients develop multifocal and bilateral papillary type 1 renal tumors. It presents a variable degree of penetrance and so far, the development of renal tumors is the only described clinical manifestation of the syndrome (8). Interestingly, patients typically present late onset tumors (sixth and seventh decades) although some reports show patients with early onset renal tumors, between the second and third decades. HPRC has been associated to an activating mutation in the c-Met oncogene on chromosome 7 (7q31). MET gene encodes the cell surface receptor for hepatocyte growth factor (HGF). MET mutation causes aberrant activity of the intracellular tyrosine kinase domain of the membrane-bound c-Met receptor, producing the cascade of activation of HGF (9).

BHD is a rare, autosomal dominant genodermatosis caused by mutations of the folliculin - codifying gene (FLCN) located on the 17p11.2. Folliculin apparently regulates the m-TOR pathway through folliculin - interacting protein (FNIP1) and 50-AMP - activated protein kinase (AMPK). Besides cutaneous lesions, affected individuals are at risk for the development of pulmonary cysts and spontaneous pneumothorax; and bilateral, multifocal kidney cancer (10). Renal tumors may present with various histologic types, and there are reports of hybrid tumors. Such histological heterogeneity is often observed within the same kidney. Mean age at diagnosis in Pavlovich et al. cohort was 51 years (11). As in VHL and HPRC patients, these tumors may be observed safely up to a size of 3cm before intervention.

Initially described in 2004 by Vanharanta et al. SDH-RCC is an inherited kidney cancer characterized by germline mutations in Krebs cycle succinate dehydrogenase B (SDHB) in patients with hereditary paraganglioma (PGL) (12). Further reports described the presence of RCC in patients with SDHB mutations either with or without a personal or family history of PGL. Later studies have also reported an association of mutations in SDH subunits C (SDHC) and D (SDHD) and inherited RCC (13, 14). The metabolic basis of HLRCC and SDH - RCC are similar and result in an early age onset, aggressive form of RCC with high metastatic potential. The enzymatic loss of function of FH might lead to a metabolic similarity with the impairment of Krebs cycle function, reliance on glycolysis, and a metabolic shift to aerobic glycolysis (14). Patients should be managed with prompt surgical intervention regardless of the tumor size. Importantly, such tumors have the potential for the development of multifocal and metachronous RCC; and nephron - sparing surgery should be considered when feasible.

TS is an autosomal dominant disorder and it affects 1 person in 6000. It is characterized by the presence of multifocal renal tumors, mental retardation, seizures and development of hamartomas in multiple organs (15). In a series of 167 TS patients, Rakowski et al. described the presence of renal lesions in 58% of patients. Of such patients, 85% had angiomyolipomas, 45% had renal cysts, and 4% had ccRCC (16). Germline mutations in TSC1 (9q34) and TSC2 (16p13.3) characterize the syndrome. Differences in the phenotype have been associated to mutations in either TSC1 or TSC2 with evidences suggesting more severe manifestations, including mental retardation and renal lesions, highly associated with mutations in TSC2 (17). TSC2 loss has been shown to result in accumulation of HIF1α and increased expression of HIF - related genes, including vascular endothelial growth factor (VEGF) (18).

CS is inherited in an autosomal dominant manner with an estimated incidence of 1 in 200.000 individuals (19). About 70% of patients with CS have germline mutations in PTEN. PTEN acts as a tumor suppressor gene through the action of its phosphatase protein product that results in inhibition of the AKT signaling pathway. Clinical manifestations include macrocephaly, multiple hamartomas, dermatologic disorders such as acral keratosis and facial tricholemmomas and increased risk for breast, endometrial and thyroid cancers. The first description associating CS and kidney cancer was made by Mester et al. who estimated that these patients had a thirtyfold increased risk of developing renal tumors of variable histology, including clear cell, papillary and chromophobe types (20). In a recent series of 24 CS patients, Shuch et al. described a 16.7% penetrance of CS - related renal tumors (21).

A recent study evaluating 82 unrelated probands with unexplained familial RCC, first described a germline mutation of BAP1 gene (BRCA1 - associated protein-1) (17). BAP1 functions as a classic two-hit tumor suppressor gene and is somatically mutated in ccRCC, uveal melanoma (22). In Farley et al. study, only one from 82 patients presented BAP1 germline mutation, however previous data described an overall germline frequency of approximately 3.8% (range of 1.9% in a group of individuals with uveal melanoma to 8.0% in a subset of apparent sporadic mesotheliomas) (17). BAP1 encodes a nuclear ubiquitin carboxyterminal hydrolase, which was initially described as binding to the BRCA1 RING finger and enhancing BRCA1 - mediated cell growth suppression (23). A later multicenter study from France evaluating families that included individuals identified as carrying germline deleterious BAP1 mutations demonstrated a significantly increased risk for RCC, which suggests that BAP1 is an RCC - predisposition gene (24).

The diagnosis of hereditary RCC syndromes can be difficult for a number of reasons such as: features with incomplete penetrance, de novo mutations, sex-specific manifestations (such as uterine leiomyomas in HLRCC), and others, such as HPRC that do not present extra-renal manifestations. Additionally, there is the need for prolonged follow-up periods for the identification of patients with bilateral or multifocal disease. The current recommendation is that all patients with bilateral or multifocal disease and patients age 46 years or younger with RCC should be referred for genetic counseling, which should be performed by an experienced team that is able to advise patients on the current clinical recommendations (Table-2).

**Table 2:** Familial syndromes related to development of renal cell neoplasia.

Syndrome	Incidence	Genes Envolved	Molecular Pathway affected	Renal Type	Others characteristics
VHL	1:36,000	VHL	Hypoxic pathway(through HIF)	Clear cell RCC	pancreatic cysts and neuroendocrine tumors, pheochromocytoma, retina I angiomas, hemangioblastomas
Birt-Hogg-Dubé	rare(unknown)	FLCN	m-TOR	Variable subtypes	cutaneous lesions, pulmonary cvsts and spontaneous pneumothorax
HPRC	rare (Iess then 1:1,500.00	MET	C-MET	Type 1 papillary RCC	not specific
HLRCC	Rare (unknown)	FH	Krebs cycle	HLRC related RCC	Multiples cutaneous and uterine leyomiomas
SDH-RCC	rare(unknown)	SDHB/SDHC/SDHD	Krebs cycle	SDH related RCC	Paragangliomas/Pheocromocytoma GIST
T5	1:6,000	T5C1/T5C2	m-TOR	Angiomyolipomas Clear Cell RCC Renal Cvsts	mental retardation, seizures and development of hamartomas in multiple organs
Cowden	1:200,000	PTEN	AKT signaling pathway	Various histologyc subtypes	macrocephalv, multiple hamartomas, dermatologic disorders such as acral keratosis and facial trichilemmomas and increased risk for breast, endometrial and thyroid cancers.

### Bladder and Renal Pelvis (Urothelial carcinoma)

Urothelial carcinomas (UC) is the 4th tumor of the urinary tract that affect specially the bladder but it can occur from renal pelvis to urethra. Among familial cancers, UC has been mainly associated to Lynch syndrome.

Hereditary non-polyposis colorectal cancer (HNPCC), also known as Lynch syndrome (LS) is an autosomal dominant familial syndrome characterized by germline mutations in mismatch repair (MMR) genes such as mutS homolog 2, colon cancer, non-polyposis type 1 (MSH2) and mutL homolog 1, colon cancer, non-polyposis type 2 (MLH1) (25). Such events lead to genetic instability and result in tumors with a high level of microsatellite instability (MSI), which is detected as alterations in short and repetitive sequences of DNA called micro-satellite regions (26). The autosomal dominant mutations are inherited with high penetrance and result in familial clustering of colorectal cancer. The extra-colonic tumor spectrum of LS includes endometrial, ovarian, urothelial, gastric, small bowel, pancreatic, hepatobiliary, brain, and sebaceous tumors. In fact, urological neoplasms represent the third most frequent Lynch-associated tumors (5%) after colonic (63%) and endometrial (9%) cancers (27).

Upper urinary tract urothelial carcinoma (UUT) is relatively rare in the normal population but patients with LS are at increased lifetime risk with an incidence as high as 6%. Ureteral carcinoma in patients with LS carries a 22% increased relative risk when compared to the risk in the general population (28). Actually, UC is considered part of the classical Lynch-syndrome tumor spectrum. Previous studies showed that the male predominance is less than that in the general population and UUT cancer is up to 7-fold more common in MSH2 than in MLH1 mutated family members (29, 30). Crockett et al. describe 39 patients with LS and UC with a predominance of ureteral tumors compared to tumors of the renal pelvis but no particular difference in pathologic parameters such as stage or histologic grade (29).

With respect to bladder cancer (BC) and LS, a number of studies have shown conflicting results. Sijmons et al. study reported a 14-fold RR (95% CI, 6.7-29.5) for UUT cancer, but the risk for developing UC of the bladder was not increased (31). While patients with MSH2 mutations have been shown to be at increased risk for UUT UC, the risk of BC in these subjects has been less investigated. Goecke et al. described a higher incidence of BC in MSH2 carriers compared with MLH1 carriers, suggesting that BC is indeed part of LS (32). Geary et al. also found a 3.6 relative risk of BC (p=0.001) in MSH2-positive compared with MSH2-negative families (30). In a recent multicenter study, Skelton et al. described a 6.21% (11/177) prevalence of BC in patients with MSH2 mutations compared with 3 of 129 patients with MLH1 mutations (2.32%) (33).

In summary, patients with LS and their relatives should be screened for UC. However, most familial UC still remains unexplained. UC seems to be a polygenic disorder, although rare familial single-gene disorders may exist.

### Prostate

Prostate cancer (PC) is the second most common tumor in men, with approximately 500.000 new cases annually in the U.S. and Europe. Despite being well studied, the etiology and pathogenesis of the disease remains poorly understood. Age, race and family history are among the known risk factors of PC. It is estimated that up to 42% of cases of PC can be assigned to familial and hereditary factors (34). First-degree relatives of men with PC present twice the risk of developing the disease. An extensive meta-analysis comprising 33 studies described that risk was greater for those men with affected brothers (relative risk [RR] 3.4; 95% CI 3.0-3.8) than for men with affected fathers (RR 2.2; 95% CI 1.9-2.5). Furthermore, the presence of two or more first degree relatives affected by PC results in higher relative risk of disease diagnosis. It is estimated that 15% of men with the disease have a first-degree relative with PC compared to 8% of the general population (35).

Unlike other types of tumors such as breast and kidney, it is suggested that the model of PC susceptibility is considerably more complex than initially thought. In fact, it is believed that multiple genes may be involved, thereby configuring what is called polygenic inheritance (36). Rare genetic events are involved with high familial risk, however due to its rarity they account for less than 5% of the total cases. Thus, in most cases there is involvement of multiple loci that confer moderate or low risk (37).

Despite extensive study, few high penetrance genes are associated with risk of developing PC. Men with mutations in BRCA1 and BRCA2 genes are known to be high-risk patients. Comparatively, the impact of BRCA1 mutation was shown to be considerably less important than BRCA2. BRCA2 mutation involves a lifetime risk of developing PC ranging from 30-40%, while BRCA1 mutation carriers present a 3–8% lifetime risk with a modestly elevated incidence of early onset prostate cancer but no increased risk in older men (38). Besides being characterized by diagnosis at earlier ages, men with BRCA1/BRCA2 mutations have high-grade tumors and more advanced stage when compared to the general population (39). Thus, this subset of patients should be managed in a specific way and strategies for prevention and treatment should be personalized.

A recent meta-analysis describes G84E germline mutation in the HOXB13 gene to be associated with a significantly increased risk of familial PC (40). HOXB13 is a homeobox transcription factor gene, which is important in prostate development. Furthermore, HOXB3 mRNA and protein are over-expressed in primary PC tissues compared to the adjacent normal prostate tissues, which suggests a potential role of HOX on prostate carcinogenesis (41). In Huang et al. study, men with PC were more likely to carry G84E allele (carrier frequency, median=1.40%; range from 0.1 to 4.9%) comparing with control subjects (carrier frequency, median=0.08%; range from 0 to 1.4%). Men with the HOXB13 G84E variant had a 4.51-fold higher relative risk of PC compared with non-carriers (95% CI 3.28–6.20). This risk effect was more pronounced in younger patients and high-grade tumors.

With the advent of new genomic tools, genome wide association studies (GWAS) have been developed, which enabled researchers to simultaneously assay up to millions of common variations in single base - pairs called single-nucleotide polymorphisms (SNPs). SNPs with a minor allele frequency >5% are called common variant alleles. GWAS have identified 76 prostate cancer risk SNPs (36). The SNPs identified are mainly in regions that had not previously been known to be associated with prostate cancer risk that might be clinically relevant, such as MYC, MSMB, KLK2 and KLK3 genes. Such SNPs explain an estimated 20% of inherited prostate cancer risk, which is highly significant when compared with breast and colon cancer (42). Recent studies have attempted to associate the presence of specific SNPs not only to the risk of developing PC but also to predict clinical outcomes and therapeutic response.

PC seems to be a polygenic disorder. The identification of patients at high risk for PC diagnosis has the potential to be a useful tool in selecting patients for screening. It is an extremely controversial issue, especially after the negative recommendation from the US Preventive Services Task Force in 2012. Incorporation of risk associated SNPs might address some of the weaknesses of PSA based screening by enabling risk stratification in screening protocols, particularly in patients who are predisposed to aggressive disease, such as BRCA2 mutation carriers. Explanation of PC inheritance will likely require an improved understanding of the interaction between these distinct genomic regions. Overall, there is limited information about benefits and harms of screening men at higher risk of PC. In addition, there is little evidence to support specific screening approaches in PC families at high risk. It is recommended that high-risk men should engage in shared decision-making with their health care providers in order to develop individualized plans for PC screening based on their risk factors.

### Testis cancer

The incidence of testicular germ cell tumors (TGCT) has steadily increased 3% to 6% annually for the last 40 years (43). Approximately, 2% of TGCT patients report an affected first degree relative. It is estimated that the relative risk of TGCT is increased 8-10-fold in siblings and 4-6-fold in sons of TGCT patients. The latter supports a role for genetic susceptibility in TGCT. The higher familial risk among brothers than father-son pairs suggest the involvement of a recessive mode of inheritance or an X-linked susceptibility locus (44).

The identification of predisposing genes has been hindered by the rarity of the disease and the wide histologic variation in TGCT presentation. GWAS of TGCT have identified eight associated SNPs at six loci, which together account for >11% of the genetic risk of TGCT (45-47). Initially, two independent genome wide association studies (GWAS) identified allele variation within KITLG on 12q22 as the strongest genetic risk factor for TGCT, with a per allele odds ratio (OR) greater than 3 (45, 48). In a recent study by Ruark et al., nine new loci for TGCT were identified, which brings the total number of TGCT-associated loci to fifteen. These fifteen loci have provided considerable new insights into TGC tumorigenesis, implicating genes involved in germ cell differentiation (DAZL, PRDM14) including the KIT-KITL signaling pathway (KITL, SPRY4, BAK1) and genes involved in sex-determination (DMRT1), microtubule assembly (TEX14, CENPE, PMF1) and telomerase regulation (TERT, ATF7IP, PITX1). The authors suggest that the nine new susceptibility alleles would account for 4% of the excess familial risk to brothers and 6% to sons of men with TGCT, which brings the cumulative totals to 15% and 22%, respectively (49).

There is strong evidence supporting a hereditary component to TGCT. However, collaborative efforts may be required to overcome the challenge posed by the rarity of this tumor.

## CONCLUSIONS

The incidence of cancer is increasing worldwide. Although population aging is in large part responsible, a more comprehensive familial risk history and advances in genomics have uncovered an increasing proportion of tumors related to cancer-syndromes. Among urinary tumors, the recognition of new entities such as SDH and HLRCC renal cell carcinomas were possible after better characterization of those syndromes.

Familiarity with the various familial phenotypes and suggestive clinical features such as early age of presentation and multicentricity should point to the possibility of a cancer related syndromes.
